# Towards digital and hands-free healthcare: exploring value co-creation interactions in eye-tracking adoption

**DOI:** 10.1108/JHOM-01-2025-0038

**Published:** 2025-11-04

**Authors:** Luis Irgang, Wiktor Zborowski, Ernesta Stakionytė, Henrik Barth, Magnus Holmén

**Affiliations:** School of Health and Welfare, Halmstad University, Halmstad, Sweden; School of Business, Innovation, and Sustainability, Halmstad University, Halmstad, Sweden

**Keywords:** Digitalization, Hands-free healthcare, Touchless interactions, Eye-tracking, Value co-creation, Technology adoption

## Abstract

**Purpose:**

Eye-tracking technology offers significant potential in healthcare by enabling hands-free interactions that reduce physical contact and infection transmission risks. However, its adoption remains suboptimal due to barriers including unfavorable cost-benefit perceptions, digital literacy gaps and workflow disruption concerns. This study explores value co-creation interactions between technology providers and healthcare professionals that facilitate eye-tracking adoption in digitalized healthcare settings.

**Design/methodology/approach:**

The study employs a qualitative approach drawing on exploratory and semi-structured interviews with eye-tracking technology providers and physicians who use eye-tracking in clinical practice and research. Data analysis follows a thematic analysis approach.

**Findings:**

The findings reveal two distinct but interconnected dimensions of value co-creation interactions that enable eye-tracking adoption. Value co-production-enabling interactions encompass aligning technology capabilities with user needs, co-developing manuals and guidelines and collaboratively interpreting regulatory frameworks. Value-in-use-enabling interactions focus on aligning technology performance with end-user experience, co-developing implementation strategies and collaborative data interpretation and analysis.

**Originality/value:**

This study contributes to healthcare digitalization literature by examining how collaborative interactions between technology providers and healthcare professionals enable complex technology adoption. Unlike previous research focusing primarily on patient-provider relationships, this study demonstrates how technology providers actively participate in healthcare value chains beyond traditional product delivery. The findings extend value co-creation theory by identifying specific interaction mechanisms within value co-production and value-in-use dimensions, contributing to understanding how collaborative approaches may support hands-free healthcare technology integration.

## Introduction

In recent years, advances in the Internet of Things, artificial intelligence, and robotics have accelerated the development of novel human-computer interfaces in healthcare. As medical devices and systems become increasingly interconnected, new opportunities emerge for hands-free control that transcends the limitations of traditional manual interaction (Klaib *et al.*, 2021; Clark and Ahmad, 2023). Among these, eye-tracking technologies are gaining momentum as they enable healthcare professionals to seamlessly operate multiple connected devices without using their hands, thereby freeing cognitive and physical resources for other critical tasks (Zhang *et al.*, 2025; Zhu *et al.*, 2024). This is particularly relevant in complex healthcare environments where efficiency and sterility are paramount. Eye-tracking reduces unnecessary physical contact by mitigating the risk of healthcare-associated infections (Bhalla *et al.*, 2021) and represents a paradigm shift in interaction design, supporting faster and more agile decision-making (Klinker *et al.*, 2020; Elhadad *et al.*, 2024). By integrating with AI-driven decision support and robotic systems, eye-tracking holds the potential to revolutionize medical practice, education, and research, thus being positioned as one of the most contemporary and promising technologies (Gu *et al.*, 2025; Tahri Sqalli *et al.*, 2023).

Eye-trackers monitor eye motion by measuring the angular position of the eye relative to the head and the orientation of the eye relative to its surroundings (Carter and Luke, 2020). Typically, eye-tracking systems consist of a device (wearable or screen-based) with multiple sensors that capture eye movement patterns, and software that records, processes, and analyzes the data generated (see Pauszek, 2023 for an overview of the functions and types of eye-tracking). These devices capture the allocation of visual attention in real-time and help identify what drives attentional behavior, thus helping to understand and predict human behavior (Duchowski, 2017). In clinical practice, eye-tracking serves as a control interface for surgical procedures by capturing surgeons’ eye movements and transmitting focus areas to integrated robotic systems (Dilley *et al.*, 2020). The technology also supports accurate diagnosis of various diseases and disorders by observing patients’ performance in visual tasks, particularly benefiting early detection of cognitive impairment (Chandrasekharan *et al.*, 2023), Alzheimer’s disease (Tadokoro *et al.*, 2021), and Autism Spectrum Disorder (Ahmed and Jadhav, 2020). Beyond clinical applications, eye-tracking offers significant advantages for hospital management by enabling performance monitoring of healthcare professionals during clinical routines (Li *et al.*, 2023; Hofmaenner *et al.*, 2021) and enhancing training effectiveness through objective assessment of visual attention patterns (Weiss *et al.*, 2023). The multifaceted applications of eye-tracking underscore its versatility in providing deep insights into human cognition and behavior, thereby enhancing hands-free interactions for more efficient and safer healthcare delivery.

Despite its benefits, the adoption of eye-tracking in healthcare is still in its infancy (Wolf *et al.*, 2023; Bapna *et al.*, 2023; Novák *et al.*, 2024), and suboptimal compared to other technologies (Iqbal and Campbell, 2021). Three main reasons account for this: first, the unfavorable cost-benefit perception creates a fundamental economic barrier. Cost has been identified as the most important organizational barrier to technology adoption in healthcare (Kumar *et al.*, 2025; Lewis *et al.*, 2022), with eye-tracking systems requiring substantial investments in customized hardware, software, and training (Ull *et al.*, 2022). The economic burden becomes more acute when considering that healthcare organizations must justify technology investment against direct patient care benefits (Kraus *et al.*, 2021), creating a challenging return-on-investment scenario for emerging technologies like eye-tracking.

Second, insufficient digital literacy among healthcare practitioners creates a critical competency gap. Studies indicate that barriers such as difficulties understanding technology, low literacy, and poor digital skills are frequently cited impediments to healthcare technology adoption (Binsar *et al.*, 2024a). This literacy gap is problematic for eye-tracking where personal and psychological barriers include healthcare professionals’ difficulty understanding the technology, technophobia, and fear of using health technologies. The steep learning curve associated with eye-tracking data interpretation complicates this challenge (Zander *et al.*, 2010; Pauszek, 2023), which may lead to misinterpretation or neglect of results (Klaib *et al.*, 2021) and hinder the effective use of eye-tracking technology.

Third, and perhaps most problematic, is that eye-tracking implementation may cause significant disruption in workflow and hospital routines, which triggers resistance to adoption. The introduction of eye-tracking technology alters established clinical workflows, information flows, and resource management patterns (Rodrigues *et al.*, 2022; Paolis, 2016), creating disturbances in deeply ingrained hospital routines that healthcare professionals rely upon for efficient patient care delivery. This disruption is particularly challenging because healthcare settings are characterized by complex organizational structures with multiple weakly-tied units embedded within larger healthcare systems, where established routines serve as critical coordination mechanisms (Irgang *et al.*, 2021; Talwar *et al.*, 2023). The resistance becomes more pronounced when eye-tracking systems require healthcare professionals to modify their visual attention patterns and decision-making processes during patient interactions or administrative tasks.

Given these adoption barriers – cost concerns, digital literacy gaps, and workflow disruption – addressing them requires collaborative approaches that leverage resources, knowledge, and competencies from multiple actors, including technology providers and vendors (Beaulieu and Bentahar, 2021; Varga and Motz, 2023). Recent studies highlight how provider-user collaboration throughout design, testing, and implementation phases directly influences adoption success, emphasizing the importance of interaction quality and real-time information exchange (Jacob *et al.*, 2022b; Kaygan and Kaygan, 2025). Evidence shows that involving clinicians in technology selection decisions significantly increases user buy-in and ownership (Bardhan *et al.*, 2023), while robust support networks and efficient after-sales support from technology providers improve adoption outcomes (Lee *et al.*, 2025). When providers demonstrate genuine interest in meeting end-user needs, healthcare professionals’ input contributes to developing user-friendly devices and enhances overall acceptance (Bird *et al.*, 2021; Zidaru *et al.*, 2021). This collaborative dynamic becomes especially critical for complex hands-free technologies like eye-tracking, which demand extensive customization, rigorous testing, and specialized training for accurate data collection and analysis (Madapana *et al.*, 2023; Tahri Sqalli *et al.*, 2023). Therefore, it is crucial to understand how strategic provider-user relationships can systematically overcome identified barriers and foster the adoption of hands-free technologies such as eye-tracking in healthcare settings.

While previous studies provide important insights into the role of relationships with multiple stakeholders as an enabler of digitalization, they primarily focus on patients, practitioners, and hospital managers’ roles in technology adoption (e.g. Iyanna *et al.*, 2022; McCarthy *et al.*, 2022; Raimo *et al.*, 2022). Research examining provider-user interaction dynamics remains limited, with studies either lacking empirical evidence (e.g. Marwaha *et al.*, 2022; Fennelly *et al.*, 2020) or presenting conflicting results about the value of such collaborations (e.g. Jacob *et al.*, 2022a; Brunner *et al.*, 2023). Although open innovation research explores collaborative partnerships (Carmona-Lavado *et al.*, 2023; Coco *et al.*, 2024), it emphasizes ideation over adoption (Xie *et al.*, 2023), while user-centered design studies focus narrowly on design or implementation phases without examining ongoing collaborative processes that ensure sustained adoption (e.g. Kujala, 2003; Yardley *et al.*, 2015). This research gap becomes particularly critical for eye-tracking technology due to its unique features: eye-tracking systems require extensive real-time data processing, specialized calibration procedures, and continuous adaptation to individual user characteristics, while providing objective measurements of gaze patterns that demand sophisticated interpretation skills (Klaib *et al.*, 2021). Unlike other healthcare technologies, eye-tracking demands highly customized interface configurations, complex integration with existing clinical systems, and ongoing technical support due to calibration challenges (Pauszek, 2023), making provider-user collaboration particularly crucial throughout the entire technology lifecycle. Given this gap in understanding stakeholder interactions in healthcare digitalization (Ruokolainen *et al.*, 2022; Balta *et al.*, 2021) and the distinctive collaborative requirements of eye-tracking technology, there is a compelling need to explore how collaborative interactions between technology providers and healthcare professionals can drive adoption of hands-free technologies such as eye-tracking.

Against this backdrop, this study aims to answer the following research question: *how do interactions between technology providers and technology end-users enable digitalization in hands-free healthcare*?

Drawing on Value Co-Creation (VCC) theory (Vargo and Lusch, 2008; Payne *et al.*, 2008), which conceptualizes provider-user interactions as creators of new value opportunities, we examine these interactions as active, collaborative processes of exchanging resources and knowledge among stakeholders (Rantala and Karjaluoto, 2016; Yu *et al.*, 2019) to create value. Through this lens, we explore the value co-creation interactions between eye-tracking providers and healthcare professionals that enable the adoption and use of eye-tracking in digitalized and hands-free routines in hospitals.

## Theoretical background

### Digitalization and technology adoption in healthcare

Digitalization represents the socio-technical transformation of products, services, processes, and business models through digital technologies, enabling connectivity across individual, organizational, and societal levels (Verhoef *et al.*, 2021). In healthcare, digitalization involves the adoption and integration of digital technologies by healthcare professionals, patients, and organizations, embedding these tools into care delivery practices and operational procedures (van Velthoven *et al.*, 2019; Stoumpos *et al.*, 2023). This transformation encompasses not only the technological infrastructure but also the organizational changes, workflow adaptations, and cultural shifts necessary to leverage technology’s full potential for improved healthcare delivery and outcomes (Kraus *et al.*, 2021; Binsar *et al.*, 2025).

The integration of technologies in healthcare creates multifaceted benefits across different stakeholder groups, fundamentally transforming how care is delivered and experienced. For patients, digitalization enhances their participation and engagement in healthcare services through improved access to health information, patient portals for appointment scheduling and test result viewing, and mobile health applications for chronic disease monitoring and medication adherence (Binsar *et al.*, 2022; Gybel Jensen *et al.*, 2024). For hospitals, digital technologies enhance organizational performance by optimizing operational processes through, for example, electronic health record systems that facilitate interdepartmental information sharing, predictive analytics for staff scheduling and inventory management, and automated workflow systems that reduce administrative burdens (Cerchione *et al.*, 2023; Binsar *et al.*, 2024b). For healthcare professionals, digital tools enhance clinical decision-making through AI-powered diagnostic assistance, clinical decision support systems for drug interaction alerts, telemedicine platforms for remote specialist consultations, and electronic prescribing systems that minimize medication errors (Jussupow *et al.*, 2021; Ismail *et al.*, 2025).

Despite these multifaceted benefits, the adoption of digital technologies in healthcare remains slower compared to other sectors (Iyanna *et al.*, 2022). A critical factor in this adoption process is the alignment between the perceived value that technologies offer, and the effort required for their implementation and sustained use (Hsieh, 2023). When this alignment is poor, healthcare stakeholders may resist adoption, underutilize available technologies, or abandon them entirely (Talwar *et al.*, 2023). The digitalization process in healthcare is further complicated by the sector’s unique characteristics, including stringent regulatory requirements, complex organizational hierarchies, and the critical nature of clinical decision-making where errors can have life-threatening consequences (Majcherek *et al.*, 2024). Unlike other industries where technology failures primarily result in financial losses or operational inefficiencies, healthcare technology failures can directly impact patient safety and clinical outcomes. This reality creates heightened scrutiny and risk aversion among healthcare professionals, who must navigate tensions between adopting innovation and ensuring patient safety (Irgang *et al.*, 2025).

Given these complexities, technology adoption models and frameworks have been extensively applied to understand why some digital innovations in healthcare achieve successful integration while others encounter implementation failures or abandonment. The Technology Acceptance Model (TAM) provides a foundational framework for understanding healthcare technology adoption by identifying perceived usefulness and perceived ease of use as primary determinants of adoption intention (Davis, 1989; Venkatesh and Davis, 2000). Perceived usefulness reflects users’ beliefs about how technology will enhance their job performance, while perceived ease of use represents expectations about the effort required for technology utilization (Holden and Karsh, 2010). These constructs can be related to the idea of functional and cost sacrifice value, where functional value represents the benefits derived from technology performance and capabilities, while cost sacrifice encompasses the monetary, time, and effort investments required for adoption and implementation (Smith and Colgate, 2007; Irgang *et al.*, 2023). Healthcare professionals thus evaluate technologies by weighing their potential to improve clinical outcomes, operational efficiency, or patient experience against implementation challenges and resource requirements (Kokshagina, 2021). The emphasis on perceived value has driven recent literature toward collaborative approaches that align technology capabilities with user needs and organizational contexts, recognizing that successful digitalization requires active engagement between technology providers and healthcare stakeholders throughout the adoption process (Jacob *et al.*, 2022b; Cohen *et al.*, 2024).

The rise of specialized hands-free technologies, such as eye-tracking systems, highlights the crucial need for close collaboration between technology providers and end-users, as these systems demand not only technical integration but also significant changes in how healthcare professionals interact with technology and interpret complex data outputs (Pauszek, 2023). These advanced technologies often require extensive customization, specialized training, and ongoing technical support that extends beyond traditional technology adoption models (Clark and Ahmad, 2023). The implementation of such sophisticated systems necessitates close collaboration between technology providers and healthcare users throughout the entire technology lifecycle, from initial development and customization to deployment, training, and maintenance (Varga and Motz, 2023). This collaborative imperative distinguishes healthcare digitalization from other sectors and evidences the importance of understanding provider-user interactions as fundamental enablers of technology integration rather than merely supplementary support activities (Balta *et al.*, 2021).

### Value co-creation in healthcare digitalization

Value co-creation (VCC) represents a collaborative process where multiple stakeholders actively engage in developing and using products or services (Vargo and Lusch, 2008; Grönroos, 2006). In healthcare digitalization, this involves combining resources, knowledge, and skills to generate both tangible and intangible value (Leone *et al.*, 2021; Spanò *et al.*, 2023). Value encompasses economic benefits (e.g. cost reduction, efficiency gains), functional benefits (e.g. improved performance, enhanced capabilities), experiential benefits (e.g. ease of use, satisfaction), and symbolic benefits (e.g. professional status, organizational reputation) that are collectively created and captured by stakeholders along the value chain (Smith and Colgate, 2007; Irgang *et al.*, 2023). While providers create potential value through their offerings, actual value emerges through practical application in clinical settings, highlighting the distinction between value-in-exchange and value-in-use (Danaher *et al.*, 2024).

Value co-creation can be understood as a process occurring across three overlapping spheres of interaction (Payne *et al.*, 2008; Grönroos and Voima, 2013). The provider sphere represents the domain where technology firms develop potential value through R&D, innovation, and solution design. In healthcare digitalization, this involves providers creating technologies and establishing technical expertise while ensuring regulatory compliance (Chatterji and Fabrizio, 2016; Tsai *et al.*, 2023). The user sphere encompasses healthcare professionals’ value-creating activities, where they integrate technologies into clinical practice to achieve specific goals such as enhanced decision-making and improved patient outcomes (Wang *et al.*, 2021). The joint sphere, where providers and healthcare professionals directly interact, represents the critical interface for value co-creation (Peng *et al.*, 2022). These interactions occur through three types of encounters: communication encounters where providers and professionals discuss technology benefits and implementation requirements, usage encounters during actual technology deployment and integration, and service encounters involving ongoing support and maintenance (Payne *et al.*, 2008). Understanding these processes is crucial as healthcare digitalization expands value creation beyond organizational boundaries (Kokshagina, 2021; Peng *et al.*, 2022).

The VCC theory has proven to be useful for exploring the dynamic interactions between multiple stakeholders aimed at the co-creation of value in healthcare. For instance, Lee (2019) examined the impact of crucial VCC elements on patients’ willingness to engage in VCC processes. The study found that the level of advanced technology, ease of access, and providers’ credibility are predictors of co-created value. Leone *et al.* (2021) conducted a case study to analyze how VCC is supported by artificial intelligence solutions. The findings revealed that AI-based service providers enhance VCC by utilizing rigorous protocols, reliable models, and a customer-centric approach. Similarly, Akter *et al.* (2022) explored the potential of shared healthcare platforms to enhance VCC. The findings indicated that patients’ and technology providers’ perception of VCC is impacted by various factors such as content-rich dialog, access to patients’ preferences, risk associated with technology usage, and the technology provider’s willingness to share transparent information with users. It is essential to note that while these studies center around VCC, they do not focus on technology adoption but rather consider technology as a tool to achieve co-creation (Masucci *et al.*, 2021). Furthermore, they primarily focus on the co-creation of value between technology providers and patients/customers, leaving a gap in evidence regarding the role of healthcare practitioners as end-users (see Supplementary Table A for an overview of representative studies).

Our study addresses this gap by exploring VCC interactions through two core conceptual dimensions: value co-production and value-in-use (Ranjan and Read, 2016). Value co-production encompasses user participation in creating and customizing solutions, while value-in-use focuses on the ongoing interactions during implementation and utilization. Understanding these dimensions is crucial for assessing how coordinated efforts between providers and users can overcome adoption barriers and ensure healthcare professionals perceive value throughout the digitalization process.

## Method

### Study design

We adopted a qualitative research approach to explore the value co-creation interactions between eye-tracking providers and healthcare professionals that enable the adoption and use of eye-tracking in digitalized and hands-free routines in hospitals. This approach provides insights into how individuals make meaning of a situation through their experiences and perceptions (Merriam, 2002), making it suitable for examining how multiple actors jointly produce value for mutual benefit (Breidbach and Maglio, 2016).

### Sampling and data collection

Data collection occurred between January and May 2022 through two stages of interviews. Using criterion and snowball sampling strategies (Palinkas *et al.*, 2015), we recruited participants based on specific criteria: (1) eye-tracking providers involved in development, commercialization, or customer support for healthcare applications, and (2) healthcare practitioners using eye-tracking devices in clinical practice or research. To identify potential respondents, we scanned online databases, manufacturer websites, discussion forums, and professional social media platforms. We sent 36 invitations to eye-tracking providers and 31 to end-users who met our criteria. Initial recruitment yielded five providers and two end-users, with snowball sampling adding one provider and two end-users. The final sample comprised six eye-tracking providers and four end-users, aligning with our focus on deep exploration rather than frequency-based analysis (Morse, 1994; Creswell, 2007).

The first stage involved three exploratory interviews with industry experts to deepen our understanding of the research phenomenon (Millar and Tracey, 2009). Building on these insights and on the literature review, we developed a semi-structured interview protocol with 10 open-ended questions based on Yu *et al*.’s (2019) interaction orientation framework, addressing interaction opportunities, quality, and resource integration. The second stage consisted of individual interviews with eye-tracking providers (*n* = 6) and end-users (*n* = 4) (Table 1). The interviews were conducted in English by the second and third authors. All interviews were conducted via video conferencing platforms, recorded, and transcribed verbatim. The respondents received the interview protocol and a description of the study’s aim in advance, and they were informed about the anonymization nature of the research and consented to being recorded.

**Table 1:** Descriptive information about the semi-structured interviews

Company	Respondent*	Formal position	Duration	Date
A	P1	Business Developer	42 min	25 April 2022
A	P2	Account Manager	34 min	13 May 2022
B	P3	CEO/Research Director	33 min	02 May 2022
C	P4	CEO	34 min	03 May 2022
D	P5	Director of Business Development	37 min	04 May 2022
E	P6	Senior R&D Developer	38 min	19 May 2022
–	U1	Neurosurgeon and Researcher	33 min	19 Apr 2022
–	U2	Neonatal Consultant and Researcher	31 min	07 May 2022
–	U3	Physician and Researcher	41 min	16 May 2022
–	U4	Physician and Researcher	32 min	23 May 2022

**Note(s):** *P = Technology Provider; U = End-user

Following the interviews, respondents were asked to share relevant documents such as reports, implementation guidelines, and sales materials. We supplemented the interview data with these secondary data sources, along with publicly available implementation guidelines and user manuals. The processes of data collection and analysis were conducted concurrently. Specifically, we engaged in the analysis of interview transcripts and relevant documents as they were obtained, rather than deferring analysis until all data collection was finalized. This iterative approach facilitated the refinement of the interview protocol, informed by emerging insights, and enriched our comprehension of the research phenomenon as the study evolved (see Supplementary Table B for additional information about the case companies and respondents).

### Data analysis

We employed thematic analysis following Braun and Clarke’s (2006) approach, with five iterative steps. First, all authors familiarized themselves with the data through repeated reading of transcripts and documents, discussing them collectively to understand the content and structure. Second, three authors independently coded the data, focusing on patterns that represented VCC interactions, defined as processes of exchanging and integrating resources between technology providers and end-users (Yu *et al.*, 2019). These interactions were analyzed in terms of their economic, functional, emotional, and symbolic value creation (Smith and Colgate, 2007; Irgang *et al.*, 2023).

Third, we collectively grouped these initial codes by similarity into second-order themes using a hybrid approach (Goel *et al.*, 2021). Fourth, all authors reviewed the coding process to identify potential new themes and verify consistency in code-theme relationships. Finally, we aggregated the codes and themes into overarching dimensions aligned with value co-production and value-in-use concepts from VCC literature (Ranjan and Read, 2016). The inclusion of secondary data sources facilitated triangulation, which is grounded on the idea of convergence of multiple perspectives to obtain corroborating evidence and attribute appropriate meanings to the data (Stake, 2010). The combination of data gathered from semi-structured interviews with supplementary documents facilitated the process of coding and refinement of themes in the thematic analysis.

**Figure 1:**
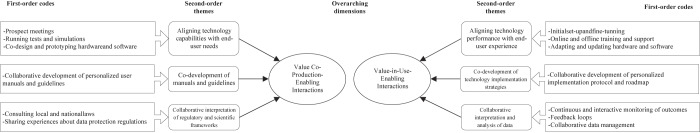
Data structure of the findings. Source: Authors’ own work

Inspired by Corley and Gioia (2004), we developed a data structure showing the relationships between first-order codes, second-order themes, and overarching dimensions (Figure 1). This structure underwent three rounds of collaborative review to ensure consensus among all authors. In the section “Findings”, we support the data structure with empirical evidence from the interviews.

## Findings

### Value co-production-enabling interactions

Value co-production-enabling interactions represent collaborations between technology providers and end-users during the pre-development and development phases of technology solutions. Technology providers orchestrate these collaborations to acquire knowledge and feedback essential for developing customized solutions that address specific healthcare challenges. For providers, value emerges through enhanced commercialization potential of their digital solutions; for end-users, value manifests in the identification of reliable providers and suitable technologies that align with their clinical needs.

Value co-production-enabling interactions fall into three categories: *aligning technology capabilities with end-user needs, co-development of manuals and guidelines*; and *collaborative interpretation of regulatory and scientific frameworks*.

*Aligning technology capabilities with end-user needs.* These interactions focus on identifying and developing technology applications that match healthcare requirements. Technology providers initiate these engagements through structured professional encounters at conferences, trade fairs, and technical visits, where they systematically gather insights about healthcare practitioners’ needs and challenges. This approach enables providers to refine their development processes while helping end-users articulate their technological requirements more effectively.

I wanted to learn whether this technology could [or could not] find any errors in risk analysis [ …] When could we use this technology? I started to attend conferences in the European conference environment. There is a research community group in that conference. I got to know providers or trackers by presenting posters. [U4]

The idea is to be involved in the research and development team, not just to buy the product [ …]. We were trying to develop the idea together [with the provider]. [U1]

These interactions also involve iterative prototyping and testing, where providers refine technology capabilities based on user feedback. For providers, this process mitigates unrealistic expectations, while for end-users, it ensures the solutions are practical and aligned with their workflows.

*Co-development of manuals and guidelines.* These interactions refer to the shared experiences and technical/scientific knowledge related to the best practices of eye-tracking devices and systems. Based on these interactions, technology providers can develop eye-tracker manuals and guidelines that are user-friendly with more accurate information.

We changed it [the user manual] last year [ …]. We organized the content to refocus, not from our engineering perspective but from the customers’ perspective. We organized it visually with a lot more space to separate the topics. We added more pictures. Our manual is less text-heavy because we now break the text with pictures. [P5]

This co-development approach not only enhances the accessibility of technical information but also facilitates the seamless integration of eye-tracking systems with complementary technologies like sensors and software. End-users value this collaborative effort, as co-developed documentation tends to be clearer and more practical than standard manufacturer manuals.

*Collaborative interpretation of regulatory and scientific frameworks*. Refer to the interactions aimed at identifying laws and regulations related to the use of eye-tracking devices and systems for collecting and analyzing human data. Technology providers must adhere to many laws and regulations in the development of eye-tracker hardware and software. Governments and regulatory organizations limit the use of eye-trackers to ensure the health and safety of patients and practitioners and to protect and control data.

[ …] there are legal contexts to protect everyone. You know about the GDPR – the General Data Protection Regulation [the European Union regulation]. In addition, we have a room at [name of location withheld] that we require our customers to use. This is our data transparency policy. [P1]

In public sector settings, these collaborations are often constrained by administrative boundaries and procurement policies. Despite these challenges, such interactions are essential for ensuring compliance and fostering trust between providers and end-users, particularly in highly regulated healthcare environments.

[ …] because we are owned by the government, we must first describe what we want; then someone else takes over the process. We are not allowed to maintain contact with manufacturers or the industry. [ …] if I want a particular instrument, I must be very precise with the specifications so that only one [the preferred] manufacturer can provide that instrument. [U4]

The value co-production-enabling interactions underscore the critical role of provider leadership in fostering pre-purchase partnerships, while acknowledging the constraints imposed by regulatory frameworks and institutional boundaries. The provider-led nature of these interactions ensures systematic technology development while maintaining alignment with healthcare requirements and compliance standards.

### Value-in-use-enabling interactions

Value-in-use-enabling interactions refer to the engagements between technology providers and end-users that facilitate the effective utilization of technology after its acquisition. These interactions ensure that technology delivers its full potential by addressing user-specific needs, fostering ongoing adaptation, and supporting seamless integration into healthcare workflows. They also strengthen relationships between providers and end-users through continuous collaboration.

Value-in-use-enabling interactions are categorized into three interrelated themes: *aligning technology performance with end-user experience; co-development of technology implementation strategies; and collaborative interpretation and analysis of data*.

*Aligning technology performance with end-user experience.* This theme focuses on tailoring eye-tracking systems to align with the practical needs and expectations of healthcare professionals. These interactions aim to identify and mitigate risks, gaps, and biases in hardware and software, ensuring that solutions meet user-specific requirements. Providers often adapt their offerings to address unexpected challenges and enhance the overall user experience.

[ …] last month someone [a physician] with a patient who had one of our devices called. The patient had a very specific eye condition that required a custom-designed keyboard from another organization we work with. We had not installed the keyboard properly when we sent the device [ …] After he called us, we worked remotely with him. We could install the specialized keyboard. [P5]

Additionally, providers often bundle services such as training, technical support, and ongoing consultation, contributing to user satisfaction and fostering long-term relationships.

We provide an “off the shelf” closed product that includes the advice and training needed throughout the product’s lifecycle. For example, we offer consultancy services in defining, setting up, and testing the device before rolling out a robust solution – either for clinical research or for commercialization. [P1].

These interactions ensure that technology is not only functional but also adaptable, ultimately increasing its acceptance and integration into workflows.

*Co-development of implementation strategies.* This theme emphasizes the collaborative efforts of providers and end-users in creating actionable plans for the integration of eye-tracking technology within organizational structures. Providers and end-users share responsibility for determining how the technology fits into existing workflows, identifying necessary changes in infrastructure, and optimizing resource flows to ensure successful implementation.

Because we help users integrate our solutions to get the best value from the eye trackers, we definitely need to work with the physicians, the nurses, and the other healthcare professionals. [P4]

Providers also engage with other stakeholders, such as IT professionals and hospital managers, to address technical and organizational challenges. Their role extends beyond installation to include monitoring and evaluating the success of implementation plans.

*Collaborative interpretation and analysis of data.* These interactions center on the joint effort to derive meaningful insights from eye-tracking data. Healthcare practitioners and technology providers engage in continuous dialog about data collection methods, analysis techniques, and interpretation approaches. This collaboration combines providers’ technical expertise with practitioners’ clinical knowledge to ensure accurate data interpretation and meaningful outcomes. Through regular consultations and shared analysis sessions, providers support healthcare professionals in developing their analytical capabilities while maintaining data quality and reliability.

I have a close business relationship with a technical person at one of our manufacturers. I share with him how I calculate my fixations and circuits [ …] because I want to make sure they aren’t wrong. [U4]

Healthcare practitioners often share access to collected data with providers, expecting their collaboration to refine data interpretation and lead to actionable insights.

[ …] we help them [the physicians and researchers] analyze the data because they don’t know how to analyze it. They collect the data and send it to us. After we analyze the data, we discuss the analysis with them. [P4]

After they [the physicians and researchers] began using it [the eye-tracker], we called them a few weeks later. We asked: “How are you doing?”, “Do you need help?” Most of them appreciate spending one or two hours with me. They show me their patient data and review what I see. In this way, they can learn more about how to use the data. [P3]

This collaborative approach facilitates continuous improvement in both data analysis methods and clinical applications. By leveraging the combined expertise of providers and users, these interactions drive continuous improvement and ensure the technology remains aligned with evolving clinical needs.

The value-in-use-enabling interactions enhance technology adoption beyond simple implementation by fostering continuous engagement between providers and healthcare professionals. As providers work alongside end-users to address operational challenges, develop targeted strategies, and optimize data analysis, they build lasting partnerships that maximize the value of eye-tracking technology in clinical practice. This collaborative approach ensures that healthcare organizations not only adopt the technology successfully but also continue to derive increasing value from their investment through sustained support and refinement of practices.

## Discussion

This study aimed to explore the value co-creation interactions between eye-tracking providers and healthcare professionals that enable the adoption and use of eye-tracking in digitalized and hands-free routines in hospitals. Drawing on value co-creation theory, we investigated how collaborative interactions between technology providers and end-users may facilitate technology integration in healthcare settings. Unlike prior research focusing primarily on patients, practitioners, and hospital managers (e.g. Iyanna *et al.*, 2022; McCarthy *et al.*, 2022; Raimo *et al.*, 2022), we examined how technology end-users interact with providers to co-create value throughout development, adoption, and use. Following Ranjan and Read (2016), we identified two dimensions of VCC interactions: value co-production-enabling and value-in-use-enabling interactions.

Value co-production-enabling interactions encompass aligning technology capabilities with end-user needs; co-development of manuals and guidelines; and collaborative interpretation of regulatory and scientific frameworks. *Aligning technology capabilities with end-user needs* represents a foundational mechanism of value co-production, where providers and healthcare professionals engage in iterative dialog to ensure technology functionality matches clinical requirements. This alignment process addresses the critical adoption barrier of unfavorable cost-benefit perceptions (Kumar *et al.*, 2025; Lewis *et al.*, 2022) by preventing investment in technologies that fail to deliver meaningful clinical value. Our findings suggest that this alignment occurs through structured professional encounters at conferences and trade fairs, followed by iterative prototyping and testing cycles that incorporate user feedback. This collaborative approach contrasts with traditional technology development models that rely primarily on market research (Postma *et al.*, 2007) rather than direct clinical input. The early involvement of healthcare practitioners in co-design activities proves crucial for identifying essential functionalities that may not be apparent to technology developers (Kaygan and Kaygan, 2025). In the context of eye-tracking applications, this alignment ensures that the complex data outputs generated by these systems can be meaningfully interpreted within existing clinical workflows. The collaborative nature of this process also helps mitigate unrealistic expectations on both sides, with providers gaining a realistic understanding of clinical constraints while healthcare professionals develop appreciation for technological possibilities and limitations.

*Co-development of manuals and guidelines* emerges as an important mechanism to bridge the digital literacy gap, a significant barrier to adopting healthcare technology (Binsar *et al.*, 2024a). Traditional manufacturer documentation, developed from engineering perspectives, often fails to translate technical capabilities into clinically meaningful guidance (Simon *et al.*, 2023). Evidence from our study indicates that collaborative documentation development transforms this technical information into user-friendly resources that bridge the gap between technological complexity and clinical application. This co-development process involves healthcare professionals sharing their experiences and providing feedback on draft materials, resulting in documentation that reflects both technical accuracy and clinical practicality. The collaborative approach ensures that manuals and guidelines address real-world implementation challenges rather than idealized use scenarios. When applied to eye-tracking systems, collaborative documentation development proves especially valuable given the steep learning curve associated with data interpretation (Zander *et al.*, 2010; Pauszek, 2023). The co-developed materials not only explain technical procedures but also provide clinical context for interpreting eye-tracking data, thus preventing the misinterpretation or neglect of results that can lead to technology abandonment (Klaib *et al.*, 2021). This mechanism also facilitates integration with complementary technologies, as co-developed documentation can address the complex interactions between eye-tracking systems and other clinical technologies.

*Collaborative interpretation of regulatory and scientific frameworks* addresses the unique challenges of healthcare technology adoption within highly regulated environments. Healthcare technology deployment must navigate complex regulatory landscapes that include data protection requirements, clinical safety standards, and institutional procurement policies (Majcherek *et al.*, 2024). Our findings indicate that successful adoption requires joint navigation of these regulatory complexities rather than providers simply ensuring compliance independently. This collaborative interpretation creates shared understanding between providers and users about regulatory requirements and their practical implications for technology use. Healthcare professionals provide insights into institutional requirements and clinical contexts, while technology providers bring expertise in regulatory compliance and technical capabilities. This collaboration ensures that regulatory compliance enhances rather than hinders technology adoption by aligning regulatory requirements with clinical needs. Regarding eye-tracking implementation, collaborative regulatory interpretation addresses critical issues such as patient data protection, consent procedures, and clinical validation requirements. The joint interpretation process also builds trust between providers and users, which is essential for successful long-term partnerships in highly regulated healthcare environments (Jacob *et al.*, 2022a).

Value-in-use-enabling interactions focus on post-purchase value creation through three mechanisms: aligning technology performance with end-user experience; co-development of technology implementation strategies; and collaborative interpretation and analysis of data. *Aligning technology performance with end-user experience* represents a dynamic process that transforms technology from a functional tool into an integrated component of clinical practice. Such performance alignment addresses the critical challenge of ensuring that eye-tracking systems not only work technically but perform optimally within the specific contexts and constraints of healthcare settings. Our findings suggest that this alignment extends far beyond initial installation to encompass continuous adaptation and customization based on real-world usage patterns. Providers actively monitor technology performance and respond to unexpected challenges, as demonstrated by cases where specialized hardware configurations were needed for patients with unique medical conditions. The bundling of services such as training, technical support, and ongoing consultation creates a comprehensive support ecosystem that enhances user satisfaction and reduces the likelihood of technology abandonment (Gillner, 2024; Dugstad *et al.*, 2019). This mechanism addresses the workflow disruption barrier identified in healthcare technology adoption literature (Rodrigues *et al.*, 2022; Paolis, 2016) by ensuring that technology integration enhances rather than impedes existing clinical routines. Eye-tracking users particularly benefit from performance alignment given the precision requirements for accurate gaze tracking and the need for seamless integration with other clinical technologies (Carter and Luke, 2020). The ongoing nature of this alignment process ensures that technology performance evolves with changing clinical needs and organizational contexts, supporting sustained adoption and utilization.

*Co-development of implementation strategies* is a collaborative process that changes technology implementation from being solely driven by the provider to a joint problem-solving effort. This approach helps minimize resistance that often arises when new technologies disrupt established workflows and organizational routines (Ismail *et al.*, 2025). Our findings indicate that successful implementation requires providers and healthcare professionals to work together in creating actionable plans that account for the complex interdependencies within healthcare organizations. This collaborative approach ensures that implementation strategies reflect both technical requirements and organizational realities, including staff scheduling constraints, patient flow patterns, and interoperability with systems and tools (Cresswell *et al.*, 2017; Esmaeilzadeh, 2022). The co-development process involves multiple stakeholder groups beyond direct users, including IT professionals, hospital managers, and administrative staff, creating a comprehensive support network for technology integration. This multi-stakeholder engagement is essential given the complex organizational structures that characterize healthcare settings, where technology adoption decisions may affect multiple departments and workflows simultaneously (van Velthoven *et al.*, 2019). Implementation of eye-tracking technologies especially requires strategy co-development as these technologies often require changes in how healthcare professionals structure their visual attention and decision-making processes during patient interactions (Ull *et al.*, 2022). The collaborative development of implementation strategies helps identify potential integration challenges early in the process, allowing for proactive problem-solving rather than reactive crisis management (Fennelly *et al.*, 2020). Additionally, this approach ensures that implementation timelines and resource requirements are realistic and achievable within existing organizational constraints.

*Collaborative interpretation and analysis of data* represents perhaps the most sophisticated mechanism of value-in-use-enabling interactions, addressing the fundamental challenge of transforming complex technological outputs into clinically actionable insights. This mechanism is particularly critical for eye-tracking technology, which generates vast amounts of complex data about visual attention patterns that require specialized interpretation skills to be clinically meaningful (Zhu *et al.*, 2024). Our findings suggest that healthcare professionals often lack the technical expertise to independently analyze eye-tracking data, while technology providers may lack the clinical knowledge to interpret data within appropriate healthcare contexts. The collaborative approach bridges this knowledge gap by combining providers’ technical expertise with practitioners’ clinical understanding, creating a synergistic relationship that enhances the value derived from eye-tracking technology. This collaboration occurs through regular consultations, shared analysis sessions, and ongoing dialog about data collection methods and interpretation approaches. These collaborative efforts address the critical barrier of data misinterpretation that can lead to non-adoption (Hsieh, 2023) by ensuring that data analysis methods are both technically sound and clinically relevant. For eye-tracking applications in clinical practice, this collaborative interpretation is essential for ensuring that gaze pattern analysis contributes meaningfully to diagnostic processes, treatment planning, and patient care decisions. The ongoing nature of these collaborative relationships ensures that data interpretation methods evolve with advancing clinical knowledge and technological capabilities, supporting continuous improvement in both analytical techniques and clinical applications.

### Theoretical contributions

This study offers contributions to the healthcare digitalization and value co-creation literature. First, our study contributes to the literature on digitalization in healthcare by examining collaborative interactions between technology providers and end-users in the context of eye-tracking adoption. While prior research has examined various stakeholder roles in healthcare technology adoption (Iyanna *et al.*, 2022; McCarthy *et al.*, 2022; Raimo *et al.*, 2022), less attention has been paid to the specific nature of provider-user collaborative interactions. Our findings suggest that understanding these interactions may be important for explaining how digital technologies are successfully integrated into healthcare practice. The identification of value co-creation interactions offers a perspective to examine how technology providers and healthcare professionals work together to address adoption challenges. Additionally, while some studies have identified the lack of perceived value as a barrier to technology adoption (Hsieh, 2023; Kokshagina, 2021), our study explores how value might be actively created through collaborative processes. This perspective differs from approaches such as open-innovation (Carmona-Lavado *et al.*, 2023; Coco *et al.*, 2024) and user-centered design (e.g. Kujala, 2003) that primarily focus on ideation and design phases rather than sustained adoption processes.

Second, our study contributes to VCC literature by examining provider-user interactions in the specific context of healthcare technology adoption. While previous VCC studies have explored various healthcare contexts (Lee, 2019; Leone *et al.*, 2021; Akter *et al.*, 2022), our research focuses specifically on interactions between technology providers and healthcare practitioners as end-users. This perspective contributes to understanding how VCC processes may function when the primary beneficiaries (patients) are not direct participants in the co-creation activities. Our findings support and extend Ranjan and Read’s (2016) framework of value co-production and value-in-use by identifying specific interaction mechanisms within each dimension. The characterization of these interactions contributes to understanding how VCC theory might apply to technology adoption contexts.

### Managerial contributions

Our findings may offer practical insights for technology firms and healthcare organizations involved in technology adoption processes. For medical technology companies, the study suggests potential strategies for engaging with healthcare professionals during technology development and implementation phases. The identified interaction mechanisms may provide guidance for organizing collaborative relationships that support technology development and diffusion. For hospital managers and procurement professionals, our findings may inform approaches to technology selection and implementation. The study suggests that identifying technology providers capable of sustained collaborative engagement may be an important consideration in procurement decisions. Additionally, understanding the types of collaborative activities that support adoption may help healthcare organizations develop more effective implementation strategies.

### Limitations and suggestions for future research

The interpretation of our research findings should be contextualized within acknowledged limitations that pave the way for future research opportunities. While our interview-based approach supports the exploratory nature of this research, we recognize methodological constraints that shaped our findings. The sample size of ten participants, while enabling detailed exploration of individual experiences and interaction patterns, inherently limits the transferability of findings across diverse eye-tracking implementation contexts, organizational structures, and healthcare specialties. Our sampling strategy, which relied on identifying participants through professional networks and referrals, likely drew us toward individuals who were already engaged with eye-tracking technology – potentially missing perspectives from those who chose not to adopt or abandoned the technology early. We also acknowledge that asking participants to reflect on past collaborative experiences may have colored their accounts. People naturally tend to rationalize their decisions and highlight positive outcomes when discussing their professional relationships, which could have influenced how they described their interactions with technology providers. The fact that we conducted interviews at a single point in time means we captured snapshots of ongoing relationships rather than observing how these collaborations evolved over months or years. Future researchers might consider following provider-user relationships longitudinally or combining interviews with direct observation of collaborative activities to build on these initial findings.

Our study, which focuses on the interactions between eye-tracker providers and end-users, leaves unexplored the perspectives of other stakeholders in the value chain. Subsequent research could enrich this understanding by integrating the viewpoints of intermediaries, patients, hospital managers, and governmental entities. A potential area for future exploration involves scrutinizing power dynamics within VCC interactions among these multiple actors, aiming to discern optimal modes for organizing and balancing power relations.

Our empirical data, drawn from a limited sample of eye-tracking providers and healthcare practitioners, represents a focused exploration of VCC interactions within a specific technological context. The findings provide insights into provider-user collaborative processes, though their broader applicability across different healthcare contexts, organizational types, and geographical regions remains to be established. Future studies should broaden the scope by including larger, more diverse samples by incorporating multiple case companies and engaging a wider range of end-users across various healthcare specialties and institutional settings. Quantitative methods could be employed to statistically validate our conceptual dimensions and test their applicability across different contexts.

VCC interactions may create a heightened dependence of end-users on technology providers. Therefore, future research should investigate strategies to mitigate this dependency while preserving co-creation benefits. Similarly, future research should investigate the effects of VCC on knowledge protection and intellectual property considerations. This is a common issue in collaborative perspectives. The exploration of open-source approaches could provide valuable insights for reducing dependence on provider resources while maintaining collaborative innovation. Additionally, future studies should develop frameworks for assessing and evaluating VCC interactions, investigating the extent to which resource and skill sharing with value chain actors proves more beneficial than traditional dyadic contractual relationships.

## Conclusion

This study explored the value co-creation interactions that enable eye-tracking technology adoption in healthcare settings, identifying two key dimensions: value co-production-enabling and value-in-use-enabling interactions. Our findings suggest that successful technology adoption may be understood as a collaborative process where perceived value is shaped through ongoing provider-user interactions. The study indicates that technology providers may play active roles in the adoption process, extending beyond product delivery to include ongoing collaboration in implementation, training, and data interpretation. By positioning healthcare professionals as active co-producers alongside providers, technology adoption may be facilitated beyond the initial implementation phase through sustained collaborative relationships. The identification of specific interaction mechanisms within value co-production and value-in-use dimensions contributes to understanding how collaborative approaches may support the successful integration of complex healthcare technologies throughout the technology lifecycle.

## Supplementary Material

Data supplement 1

Data supplement 2

## Notes

The supplementary material for this article can be found online.
