# Supplementary material for: Towards digital and hands-free healthcare: exploring value co-creation interactions in eye-tracking adoption
Source: J Health Organ Manag. 2025 Nov 4;39(9):498–520. doi: 10.1108/JHOM-01-2025-0038 (PMC12596749; doi:10.1108/JHOM-01-2025-0038)
Supplement: Data supplement 1 [file jhom-01-2025-0038_suppl1.docx]

**Supplementary Table A.** Representative studies of VCC interactions in technology adoption.

| **Authorship and journal** | **Objectives/Research questions** | **Method** | **Key Actors Involved** | **Theoretical Approach** | **Key insights on VCC interactions** |
| --- | --- | --- | --- | --- | --- |
| Cohen et al. (2024), *The American Journal of Managed Care* | To understand the role of health information technology (IT) vendors and health IT functionality in supporting advanced primary care | Longitudinal study: 203 interviews with healthcare practitioners and managers, and 15 health IT vendors and policy experts | Healthcare practitioners and managers, and IT vendors | Consolidated Framework for Implementation Research | Interactions governed by formal contracts enhance expectation management, facilitate customized support to users, increase vendors’ commitment to provide support and meet quality, usability, and legal requirements, and assist vendors with predicting the provision of services |
| Gillner (2024), *Social Science & Medicine* | To understand how providers of AI-enabled diagnostics perceive and navigate the spread of AI in complex healthcare systems | 17 interviews with AI providers and healthcare managers and practitioners | AI providers | Complexity Science | - Interactions between technology providers and end-users must be based on agile practices - Providers must adjust their communication strategies to alleviate the implementation pressure on physicians and reduce uncertainty and fear - Interactions must reduce tensions and the cultural distance between technology provider and adopting organization |
| Brunner et al. (2023), *Journal of General Internal Medicine* | To identify perceived impacts of adopting single-vendor, integrated EHR systems on the institutional EHR workforce | Interviews with 42 healthcare and IT professionals from four healthcare systems | Healthcare practitioners and IT staff | Organizational Transition | - The necessity of reaching out to vendors can foster a sense of reduced autonomy among healthcare professionals - Strict contracts may limit flexibility in vendor relationships, potentially leading to demotivation among healthcare staff - Inefficient communication channels can hinder effective dialogue with vendors |
| Lewis et al. (2022), *Healthcare Informatics Research* | (1) To assess current data management and security procedures; (2) to identify attitudes, knowledge, perceived norms, and self-efficacy regarding the adoption of advanced cryptographic techniques; and (3) to offer guidelines that could help policymakers and data security professionals work together to ensure that patient data are both secure and accessible | 12 semi-structured interviews with healthcare managers | Healthcare managers | - | - The interactions between users and technology developers must be planned and coordinated. - Vendors must convince healthcare organizations that their products can offer added value over existing solutions and will be a worthwhile investment |
| Esmaeilzadeh (2022), *Technology in Society* | (1) To identify and categorize the main barriers to using HIE in healthcare organizations; and (2) to develop a comprehensive model using the identified inhibitors to explain and predict how these barriers may affect clinicians’ intention to engage in HIE networks | Survey with 318 clinicians from US hospitals | Physicians | Utility theory | Effective collaboration encompasses:   - Ensuring the inclusion of detailed contractual agreements, including warranties, payment schedules, vendor responsibilities, cost quotations for the system, and ongoing system support - Co-designing a technical strategy aimed at fostering system interoperability - Cautioning vendors against exerting coercive pressures on healthcare organizations to acquire additional tools or solutions for achieving interoperability - Acknowledging that vendor-imposed fees can hinder the implementation of the intended system |
| Jacob et al. (2022), *JMIR Human Factors* | (1) To understand the challenges and opportunities in the inclusion of patients in the development of eHealth technologies and ideas on how to overcome the identified gaps; and (2) to create a research-based end-to-end practical blueprint that can guide the relevant stakeholders to successfully engage patients as cocreators in all human-center design phases | 20 interviews with multiple stakeholders (eHealth experts, Health insurance experts, Healthcare professionals, patient advocates, patient experts, pharmaceutical executives, technology providers) | Patients | Human-centric design | To foster trust and advocacy, the interactions between providers and patients must involve embedding users in the technology development team, managing expectations from the beginning to avoid frustration, and relying on engagement agencies for recruitment and professional moderation to improve communication. |
| Marwaha et al. (2022), *NPJ Digital Medicine* | To discuss the dimensions along which health systems should evaluate digital health tools to anticipate the potential benefits and challenges of adopting them | Conceptual paper | - | - | - Shared responsibility between vendors and users for identifying needs related to maintenance, updates, and new features - Vendors offer scientific support by providing evidence of the tool's efficiency - Interactions involving data sharing are probable, with a crucial requirement for anonymizing the shared data - Interactions are regulated by formal contracts - Users are assured the right to updates and maintenance, even in the event of the provider being acquired or going out of business |
| Fennelly et al. (2020), *International Journal of Medical Informatics* | To summarize the findings from literature reviews with a view to identifying and exploring the key factors which impact on the success of an EHR implementation across different healthcare contexts | Umbrella review of 27 literature reviews. | Multiple stakeholders | Implementation theory | - Implementation groups at hospitals should cultivate strong, trusting relationships with vendors - Co-designing the implementation strategy with clear, measurable objectives, an appropriate implementation process, and defined roles and responsibilities - Vendors should demonstrate openness to sharing code development data and willingness to adapt their product to meet the users’ needs |
| Dugstad et al. (2019), *BMC Health Services Research* | To identify the facilitators and barriers for implementation of digital monitoring technology in residential care for persons with dementia and wandering behavior, and explore co-creation as an implementation strategy and practice | Longitudinal case study: 7 workshops and 23 interviews with healthcare practitioners and managers, and technology vendors | Healthcare practitioners and vendors | Measurement instrument  for determinants of innovation (MIDI) framework | - Interactions in pre-implementation stage must include stakeholder mapping, as well as identifying potential implementation risks and incompatibility with existing and new technology - Cultural differences (healthcare professionals X vendors) may delay the implementation process - Knowledge translation must bridge the gap between users and IT experts - Regular workshops and establishment of “fast-working, self-dissolving task-teams” to solve implementation problems - Formation of coalitions with implementation champions/superusers helps to sustain the use of the new technology - Establishment of routine of daily feedback on the functionality of the technology right after the implementation - Assessment of usability of technology through workshops and observations - Development of mutual trust and a constructive dialogue |
| Cresswell et al. (2017), *Health Services Research* | To explore and understand approaches to user engagement through investigating the range of ways in which healthcare workers and organizations accommodated the introduction of computerized physician order entry and computerized decision support for hospital prescribing | Longitudinal case studies: 24 observations and 173 interviews with healthcare professionals | End-users (healthcare professionals) | - | Interactions must elevate user experience by:   - Encouraging end-users to report perceived usability issues as they arise - Facilitating innovation pathways for the seamless integration of new ideas into system design - Recognizing the willingness and ability of vendors to collaborate as a key factor in promoting user feedback and innovation |
