# Supplementary material for: Towards digital and hands-free healthcare: exploring value co-creation interactions in eye-tracking adoption
Source: J Health Organ Manag. 2025 Nov 4;39(9):498–520. doi: 10.1108/JHOM-01-2025-0038 (PMC12596749; doi:10.1108/JHOM-01-2025-0038)
Supplement: Data supplement 2 [file jhom-01-2025-0038_suppl2.docx]

**Supplementary Table B.** Descriptions of the case companies and respondents.

| **Technology Provider/**  **End-user** | **Description** |
| --- | --- |
| Company A  (Sweden) | Company A provides services and products that assist researchers in developing discoveries in various fields such as psychology and neuroscience, marketing, human performance, and training. In healthcare settings, the company offers healthcare practitioners training and simulations through eye-tracking devices. |
| Company B  (The Netherlands) | Company B works with a range of healthcare practitioners and machine learning specialists to incorporate novel technological and scientific advances in healthcare for children and adults with eye movement issues. The company offers a device that, when connected with an eye-tracker and a platform, can automatically assess any eye movement and provide robust solutions. |
| Company C  (France) | Company C is a specialist in preventive clinical diagnosis that employs machine learning models to analyze eye movement data acquired with cutting-edge, eye-tracking technology. The company provides technology solutions to physicians, patients, and healthcare and research centers. |
| Company D  (USA) | Company D develops an inconspicuous human-computer interface that transforms how people interact with devices. The company’s products and services are applied in various fields, including scientific research, marketing, usability testing, simulations, and assistive technology. |
| Company E  (Austria) | Company E is a specialist in providing eye-tracking solutions for manufacturing, research and analysis, security, and healthcare. The company offers hands-free eye-tracking solutions for hospitals, especially for use by operating room personnel and in education and training for healthcare professionals. |
| End-user U1  (Italy) | End-user U1 is a healthcare professional in neurosurgery at hospitals. Currently, in collaboration with Company A, this end-user is developing a novel technique in neurosurgery that helps track a patient's eye movement after surgery. |
| End-user U2  (Germany) | End-user U2 is a neonatal consultant in charge of a simulation training center where regular training is provided for physicians, nurses, and midwives, and research is conducted that focuses on simulations, human factors, and patient safety. An eye-tracking device is used daily to analyze human factors in simulating clinical events. |
| End-user U3  (Israel) | End-user U3 is a highly experienced physician and researcher. Respondent uses eye-tracking technology in laser surgery for glaucoma, lazy eye treatment, and in diagnosing schizophrenia or other diseases. |
| End-user U4  (Norway) | End-user U4 is a highly experienced physician and researcher currently employed as an associate professor. Respondent uses eye-tracking technology in research related to children, special vision development, and binocular vision. |
